# Editorial: Structural immunology of molecular innate immunity

**DOI:** 10.3389/fimmu.2023.1284121

**Published:** 2023-09-06

**Authors:** Jun Hyuck Lee, Qian Yin, Hyun Ho Park

**Affiliations:** ^1^ Unit of Research for Practical Application, Korea Polar Research Institute, Incheon, Republic of Korea; ^2^ Department of Biological Science, Florida State University, Tallahassee, FL, United States; ^3^ College of Pharmacy, Chung-Ang University, Seoul, Republic of Korea

**Keywords:** cGAS-STING, complement receptors, innate immunity, structure, TLR

In the early stages of the host defense response, innate immunity plays a crucial role due to its ability to promptly recognize pathogenic or danger signals through various receptors located on cell surfaces or within the cytoplasm (1). This initial recognition event is subsequently followed by signal transduction processes involving a range of adaptor and effector molecules. The primary goals of the innate immune system include identifying and eliminating invading pathogens, recruiting diverse immune cells to the infection site, and facilitating preparations for the adaptive immune response (2). Over several decades, extensive investigations have been conducted to unravel the intricacies of the innate immune system, leading to an increasingly detailed comprehension of its molecular foundations (3–5).

The Research Topic “*Structural Immunology of Molecular Innate Immunity*” highlights 13 recent studies that delve into the process of innate immunity at the molecular level, with a specific focus on toll-like receptor (TLR)-mediated immune responses, cGAS-STING-mediated innate immunity, GTPase-mediated antiviral processes, the structural biology of the complement system, and Janus kinase as a drug for inflammatory diseases.

Toll-like receptors (TLRs) are well-established pattern recognition receptors responsible for recognizing pathogens and triggering innate immune responses. Since their discovery, TLRs have ushered in a new era in immunology, bridging the gap between initial pathogen recognition by innate immune cells and the subsequent initiation of the adaptive immune response. Recent investigations have further revealed that TLR signaling directly controls T cell activation, proliferation, differentiation, developmental processes, and overall function, covering a wide array of physiological scenarios. Duan et al. reviewed the multifaceted roles of TLR signaling, both through direct and indirect mechanisms, in governing cell-mediated immunity. They discussed the significance of TLR signaling in the host’s defense mechanisms against a spectrum of challenges, including infectious diseases, autoimmune disorders, and cancer. Another group, Zheng and Dong, also reviewed the role of TLRs in pathogen detection *in vivo*. They summarized recent findings of TLRs’ contributions to the differentiation of naïve CD4+ T cells into T helper (Th) cells, initiation of immune responses, and active participation in the pathogenic mechanisms underlying autoimmune and allergic disorders.

A couple of research groups reported original research in the field of TLR biology. Wildum et al. evaluated the therapeutic effect, host immune reactions, and safety of RG7854, an oral double prodrug of a toll-like receptor 7 (TLR7) agonist, for developing medication for chronic hepatitis B (CHB). Based on their evaluation, they concluded that TLR7 agonists hold promise as immunotherapeutic avenues for achieving a functional cure in CHB patients. Jiang et al. highlighted the important role of surfactant protein D (SP-D), a well-known innate immune molecule. This study revealed that SP-D engages with the TLR4/MD-2 complex to suppress TLR4-mediated PI3K/Akt and NF-κB signaling activation within chondrocytes. These insights underscore the chondroprotective attributes of SP-D, relying on TLR4-mediated PI3K/Akt and NF-κB signaling pathways.

This Research Topic also includes four papers on cGAS-STING, a sensing axis related to innate immunity that has recently garnered significant attention. Cyclic GMP-AMP synthase (cGAS) identifies double-stranded DNA (dsDNA) from invading pathogens, initiating an interferon response by activating the downstream adaptor protein stimulator of interferon genes (STING). This represents the classic and fundamental biological role of the cGAS-STING signaling pathway, crucial for preventing the invasion of pathogenic microorganisms. Additionally, cGAS can interact with various types of nucleic acids, such as cDNA, DNA: RNA hybrids, and circular RNA, contributing to a diverse range of biological functions. A growing body of research has unveiled a significant connection between the cGAS-STING signaling pathway and autophagy, cellular senescence, antitumor immunity, inflammation, and autoimmune diseases. Wang et al. thoroughly reviewed the action mechanism of cGAS as it interacts with different types of nucleic acids, its multifaceted biological functions, and the potential for targeting this pathway to treat various diseases. Shi et al. conducted an evaluation of research on the cGAS-STING pathway, predicting the hotspots and emerging trends in the field using bibliometric analysis. This analysis revealed a shift in research focus from understanding how cGAS senses dsDNA and how cGAMP binds to STING to exploring the roles of the cGAS-STING pathway in various pathological states

The global prevalence of obesity, prediabetes, and diabetes is increasing, and these metabolic disorders are closely intertwined with neurodegenerative conditions, particularly Alzheimer’s disease and related dementias. The connection between these disorders is shaped by innate inflammatory signaling, potentially involving the early activation of the cGAS/STING pathway. Elzinga et al. uncovered acute systemic changes in metabolic and inflammatory responses, marked by impaired glucose tolerance, insulin resistance, and shifts in the composition of peripheral immune cell populations. In the central nervous system, noticeable inflammatory changes manifested as microglial activation within a pro-inflammatory environment, occurring concurrently with cGAS-STING pathway activation. Notably, experiments involving neuron-microglial co-cultures demonstrated that blocking gap junctions led to a substantial reduction in cGAS-STING activation. The research also reported on the precise downstream signaling events of the cGAS-STING pathway. Xia et al. systematically analyzed porcine STING (pSTING), revealing its capability to induce IFN, autophagy, and apoptosis. The investigation unveiled distinct dynamics governing the three downstream events associated with pSTING, underscoring the emergence of IFN-independent triggers for autophagy and apoptosis. Furthermore, the study explored the regulatory influence of autophagy on pSTING-induced IFN and apoptosis.

The significance of GTPases in innate immunity has been highlighted by a couple of research groups. Zhang et al. presented their original research article concerning the function and mechanism of grass carp ADP ribosylation factor 1 (gcARF1) in viral infection. They confirmed that the small GTPase domain of gcARF1 is essential for promoting grass carp reovirus (GCRV) replication and infection, with the inhibition of gcARF1 GTPase activity resulting in a significant reduction in the number of VIBs. Their study demonstrated the indispensable role of gcARF1’s GTPase activity in facilitating efficient GCRV replication. Ha et al. provided insights into the activation mechanism of immunity-related GTPase B10 (IRGB10) by presenting the crystal structures of GppNHp-bound and nucleotide-free IRGB10. These structures revealed that IRGB10 exists as a monomer in both nucleotide-free and GTP binding states. Furthermore, they determined that GTP hydrolysis plays a critical role in dimer formation and subsequent oligomerization of IRGB10. Building upon these findings, they proposed a mechanistic model to explain the operational mechanism of IRGB10 during the disruption of pathogen membranes.

The complement system assumes pivotal roles in a diverse array of immune and inflammatory processes and is often implicated as a causative or exacerbating factor in various human ailments, ranging from asthma to cancer. Complement receptors consist of no fewer than eight proteins categorized into four structural classes. These receptors coordinate complement-mediated humoral and cellular effector responses, while concurrently orchestrating intricate communication between the innate and adaptive arms of immunity. Santos-Lopez et al. reviewed a comprehensive overview of the current understanding of the structural biology underpinning complement receptors and their interactions with natural agonists and pharmacological antagonists. The review accentuates fundamental principles and delineates the unresolved areas where challenges and uncertainties persist, including the identification of research gaps. In the field of the complement system, Navas-Yuste et al. reported a structural study. They successfully deciphered the X-ray crystallographic structure of the glyceraldehyde-3-phosphate dehydrogenase (GAPDH) enzyme from L. interrogans. GAPDH is an enzyme involved in glycolysis and has been shown to serve additional functions in various pathogenic organisms, bolstering their infectivity and immune evasion capabilities. With this structural information, they established the interaction between L. interrogans GAPDH and the human innate immune anaphylatoxin C5a through *in vitro* experiments. These findings suggest L. interrogans GAPDH as a potential immune evasion factor targeting the complement system. Additionally, Kwon provided a review on Janus kinase (JAK). The Janus kinase (JAK) family of enzymes represents a class of non-receptor tyrosine kinases crucial for phosphorylating cytokine receptors and signaling transducer and activator of transcription (STAT) proteins within the JAK-STAT signaling pathway. JAKs have emerged as attractive drug targets to counteract aberrant JAK-STAT signaling. This review delves into the multifaceted roles of JAKs within the JAK-STAT signaling pathway, providing an in-depth analysis of JAK structures and their conformational alterations critical for catalytic activity.

While advanced biological techniques have unveiled the molecular understanding of innate immunity, there is still much to learn about the molecular processes underlying innate immunity. In general, this Research Topic presents timely articles highlighting the current understanding of innate immunity at the molecular level. The authors express their gratitude to all contributors for sharing their discoveries and to the referees for their careful and insightful reviews. It is believed that the included articles will be of great interest to researchers studying innate immunity at the molecular level.

## Author contributions

JL: Conceptualization, Writing – original draft. QY: Conceptualization, Writing – original draft. HP: Conceptualization, Funding acquisition, Project administration, Supervision, Writing – original draft, Writing – review & editing.
